# Cefiderocol-resistant pathogens in German hospital wastewater: a reservoir for multidrug resistance

**DOI:** 10.1038/s41598-025-17379-2

**Published:** 2025-08-27

**Authors:** Tim Erler, Laura Carlsen, Jennifer Dengler, Jens Andre Hammerl, Andreas J. Stroehlein, Marc Hoffmann, Johannes K. Knobloch, Christoph Lübbert, Cihan Papan, Thomas Schwanz, Janine Zweigner, Anurag Kumar Bari, Basil Britto Xavier, John W. A. Rossen, Nico T. Mutters, Mykhailo Savin

**Affiliations:** 1 Institute for Hygiene and Public Health, University Hospital Bonn, Venusberg-Campus 1, 53127 Bonn, Germany; 2 Institute of Medical Microbiology, Virology, and Hygiene, Department for Infection Prevention and Control, University Medical Center Hamburg-Eppendorf, Hamburg, Germany; 3 Integrative Health and Security Management Center, Staff Section Environmental Protection and Sustainability, Jena University Hospital, Jena, Germany; 4 Department for Biological Safety, German Federal Institute for Risk Assessment, Berlin, Germany; 5 Division of Infectious Diseases and Tropical Medicine, Department of Medicine I, Leipzig University Hospital, Leipzig, Germany; 6 Division of Infection Control and Infectious Diseases, University Hospital RWTH Aachen, Aachen, Germany; 7 Department of Hospital Hygiene and Infection Control, University Hospital of Cologne, Cologne, Germany; 8 Department of Medical Microbiology and Infection Control, University of Groningen, University Medical Center Groningen, Groningen, The Netherlands; 9 Laboratory of Medical Microbiology and Infectious Diseases & Isala Academy, Isala Hospital, Zwolle, The Netherlands; 10 Department of Pathology, University of Utah School of Medicine, Salt Lake City, USA

**Keywords:** Pandrug-resistant bacteria, Cefiderocol resistance, Multidrug-resistant pathogens, Wastewater surveillance, Plasmid incompatibility types, Carbapenemases, Antimicrobial resistance, Policy and public health in microbiology, Clinical microbiology, Water microbiology

## Abstract

Cefiderocol-resistant bacteria pose a growing concern in both clinical and environmental settings. This study investigates cefiderocol-resistant bacteria in wastewater from six German tertiary care hospitals. A total of 36 samples were analysed using a culture-dependent approach involving cefiderocol pre-enrichment, yielding 97 cefiderocol-resistant isolates—primarily *Enterobacter roggenkampii*, *Klebsiella oxytoca*, *Serratia marcescens*, and *Citrobacter farmeri*. Most isolates exhibited high minimum inhibitory concentrations against cefiderocol and resistance to multiple antibiotics. Resistance rates were lower for meropenem-vaborbactam (10.3%) and imipenem-relebactam (33.0%), while all isolates remained susceptible to aztreonam-avibactam. Whole-genome sequencing of 79 isolates revealed a diverse resistome, with 78.5% (62/79) carrying carbapenemase genes. Some isolates harbored up to six distinct β-lactamase genes, including combinations of extended-spectrum β-lactamases (ESBLs), AmpC β-lactamases, and one or multiple carbapenemases—such as as *bla*_OXA−48_ co-occurring with *bla*_NDM−1_ or *bla*_VIM−1_. In addition, biocide and heavy metal resistance genes were prevalent, highlighting bacterial adaptation to harsh environments. Plasmid profiling showed significant interspecies variation, with *C. farmeri* and *K. oxytoca* displaying the highest plasmid loads. Across all isolates, 38 unique plasmid incompatibility types were detected, 18 of which were species-specific. These findings highlight the multidrug-resistant nature of wastewater-derived pathogens and the importance of monitoring resistance dissemination in healthcare environments.

**Supplementary Information:**

The online version contains supplementary material available at 10.1038/s41598-025-17379-2.

## Introduction

Multidrug-resistant (MDR) Gram-negative bacteria, such as carbapenem-resistant *Enterobacterales*, *Pseudomonas aeruginosa*, and *Acinetobacter baumannii*, present a critical threat to public health, especially within healthcare settings^1^. Critically ill patients are especially vulnerable due to their compromised immune systems, increasing the risk of severe and difficult-to-treat infections^1,2^.

In 2017, the WHO designated key pathogens such as *Pseudomonas aeruginosa*, *Acinetobacter baumannii*, *Klebsiella pneumoniae*, and *Enterobacter* species as “priority pathogens” within the ESKAPE group, emphasizing the urgent need for new antibiotic developments. In the most recent Bacterial Priority Pathogens List, carbapenem-resistant *Enterobacterales* and *A. baumannii* remained in the highest risk category, reaffirming their classification as critical priority pathogens^1^. Notably, *A. baumannii* exhibited carbapenem resistance rates of 50% or higher in 56% of EU/EEA countries, predominantly in southern and eastern Europe. In contrast, Germany reported significantly lower carbapenem resistance rates, with *P. aeruginosa* showing the highest resistance at 14.8%, followed by *Acinetobacter* spp. at 4.3%^2^.

To address these resistance challenges, the medical community has developed innovative antimicrobial agents with unique mechanisms of action. These include novel β-lactam–β-lactamase inhibitor (BL-BLI) combinations (e.g., meropenem-vaborbactam, imipenem-relebactam), the next-generation tetracycline eravacycline, and the semi-synthetic aminoglycoside plazomicin. However, gaps remain in their efficacy^3–5^. Consequently, healthcare providers often resort to using polymyxins (i.e., colistin), despite their known risks and suboptimal clinical outcomes in critical patient populations^6^.

Amidst these challenges, cefiderocol has emerged as a ground-breaking synthetic siderophore-conjugated antibiotic^7^. Its unique chemical structure enhances β-lactamase stability while conferring potent siderophore activity, allowing cefiderocol to effectively overcome multiple β-lactam resistance mechanisms, including β-lactamase production, porin mutations, and efflux pump activity. Approved by the Food and Drug Administration (FDA) in 2019 for hospital-acquired and complicated urinary tract infections, cefiderocol is also endorsed by the European Medicines Agency (EMA) for treating aerobic Gram-negative infections in adults with limited treatment options^8^.

As of 2020, cefiderocol is advised for use in treating both hospital-acquired bacterial pneumonia and ventilator-associated bacterial pneumonia that are caused by Gram-negative microorganisms^7^. In Germany, cefiderocol is used as a reserve antibiotic for the treatment of infections caused by multidrug-resistant aerobic Gram-negative bacteria, particularly when therapeutic options are limited. This includes pathogens that are frequently carbapenem-resistant or carbapenemase-producing, and/or exhibit resistance to multiple key antibiotic classes such as β-lactams (ureidopenicillins, third- and fourth-generation cephalosporins, carbapenems) and fluoroquinolones^9^.

In this study, recent data from participating university hospitals in Germany illustrate the cautious yet targeted use of cefiderocol in clinical settings. One center reported average consumption levels of 0.03 and 0.04 RDD/100 bed-days for the years 2023 and 2024, respectively. At another site, cefiderocol usage between 2021 and 2024 was documented in 25 individual patients, with pharmacy records indicating an average of 0.01 to 0.05 RDD/100 bed-days. A third hospital noted that cefiderocol administration was predominantly restricted to high-risk wards, including intensive care, infectious diseases, gastrointestinal, and surgical units. These findings highlight that cefiderocol is predominantly employed in critically ill patients where few alternative treatment options remain.

The introduction and use of cefiderocol in treating carbapenem-resistant or carbapenemase-producing Gram-negative pathogens mark a significant breakthrough in the field of infectious disease management^9^. Its effectiveness against bacteria that are resistant to key antibiotic classes provides a crucial treatment option for severe infections that are otherwise difficult, if not impossible, to treat. Consequently, cefiderocol plays an essential role in combating the public health challenge posed by antibiotic-resistant bacteria, offering hope in situations where limited therapeutic alternatives exist. However, the recent emergence of cefiderocol resistance among clinical pathogens, as reported in various studies, underscores the need for continued surveillance^10,11^. According to Karakonstantinis et al.. (2024), cefiderocol non-susceptibility was alarmingly high among New Delhi metallo-β-lactamase (NDM)-producing *Enterobacterales* (38.8%), NDM-producing *A. baumannii* (44.7%), and ceftazidime-avibactam-resistant *Enterobacterales* (36.6%)^11^. In this study, resistance data from participating university hospitals further support the need for close monitoring. At one site, phenotypic resistance to cefiderocol was confirmed in 89 of 128 tested carbapenem-resistant clinical isolates (69.5%) in 2023 and in 121 of 158 isolates (76.6%) in 2024. Another participating hospital reported cefiderocol resistance among carbapenem-resistant clinical isolates (excluding screening samples) in 2024, with resistance rates of 67% in *E. coli* and *Enterobacter* spp., 50% in *Proteus* spp., and 43% in *Klebsiella* spp. In a third institution, approximately 50% of patients transferred from Ukraine developed secondary resistance during ongoing cefiderocol therapy, and primary resistance was already present in several cases upon admission. These observations point toward an emerging threat of resistance even to this last-line antibiotic, particularly in high-risk patient populations.

Clinical wastewater, often considered a reservoir for nosocomial high-risk clones, serves as a convenient and ethically appropriate monitoring matrix and may be well-suited for the surveillance and epidemiology of cefiderocol-resistant bacteria within hospital settings. Thus, in the present study we provide insights into the occurrence and diversity of cefiderocol-resistant bacteria of clinical relevance in wastewater from maximum care hospitals representing different endemic regions in Germany. Furthermore, we characterize the isolated target bacteria by analysing their phenotypic and genotypic antimicrobial resistance profiles, including resistance to biocides and heavy metals, as well as their virulence properties and plasmid diversity. The study is crucial for understanding both cefiderocol resistance in healthcare settings and the role of wastewater systems as conduits for their spread. Its findings are expected to inform public health strategies to effectively tackle antimicrobial resistance, highlighting its critical role in protecting public health.

## Results

### Cefiderocol-resistant bacteria in wastewater: occurrence and resistance profiles

Cefiderocol-resistant bacteria were identified in 50% of wastewater samples (18/36), with detection rates varying significantly by location: from 16.7% (1/6 samples) at TCH6 to 66.7% (4/6 samples) at TCH1 and TCH2. A total of 97 cefiderocol-resistant isolates were recovered and identified, with the most prevalent species being *E. roggenkampii* (*n* = 44), *K. oxytoca* (*n* = 16), *S. marcescens* (*n* = 12), *C. farmeri* (*n* = 10), and *K. michiganensis* (*n* = 8). Additional species were identified in smaller numbers, including *Enterobacter asburiae* (*n* = 3), *Enterobacter soli* (*n* = 1), *Myroides odoratus* (*n* = 1), *K. pneumoniae* (*n* = 1), and *Elizabethkingia anopheles* (*n* = 1). Most isolates exhibited high MICs against cefiderocol, with 80 out of 97 isolates showing MICs ≥ 32 mg/L, 15 with MICs of 16 mg/L, and only two isolates—*K. pneumoniae* and *E. anopheles*—displaying MICs of 4 mg/L.

**Fig. 1:**
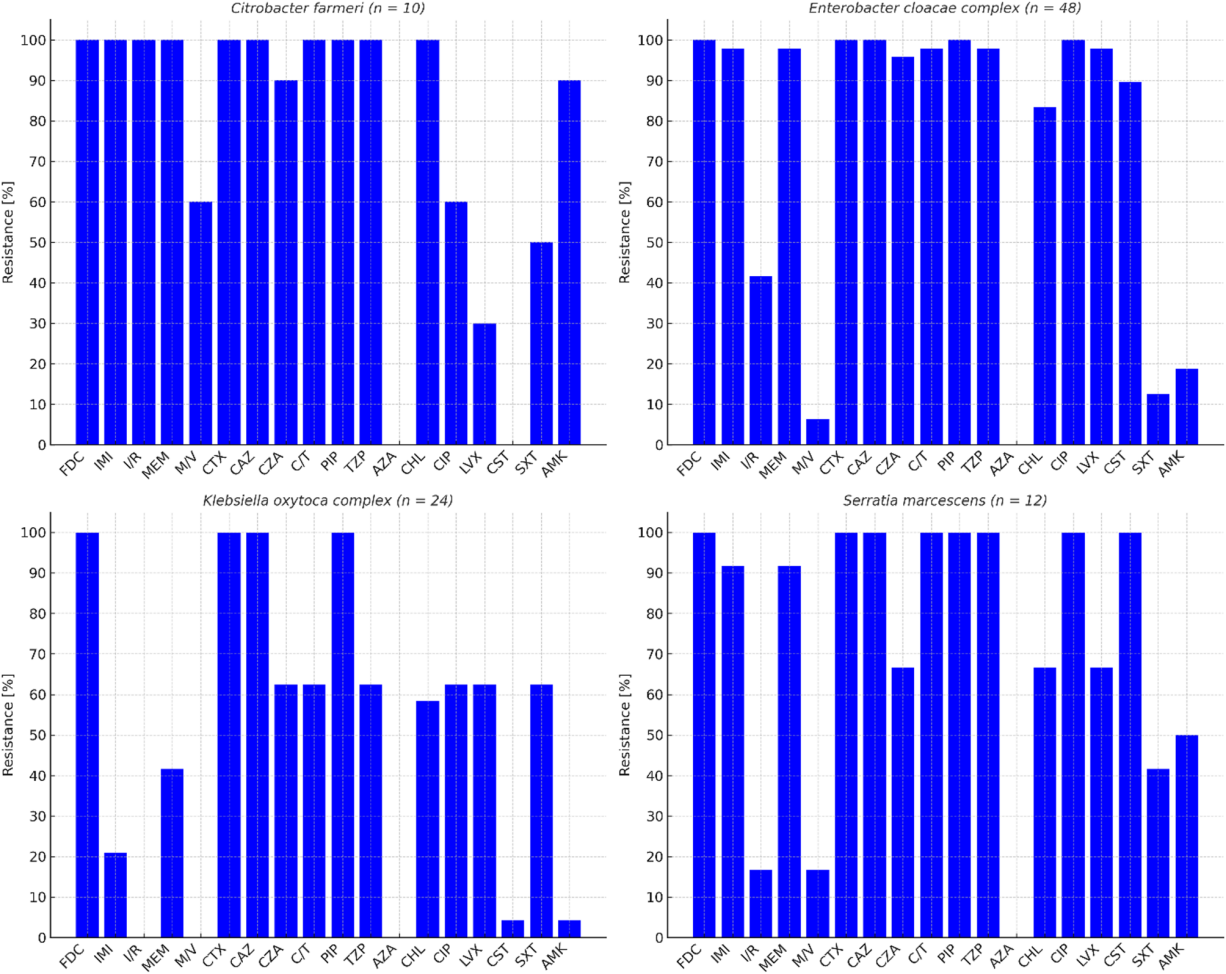
Phenotypic antimicrobial resistance detected in cefiderocol-resistant isolates of *Citrobacter farmeri* (*n* = 10), *Enterobacter cloacae* complex^a^ (*n* = 48), *Klebsiella oxytoca* complex^b^ (*n* = 24), and *Serratia marcescens* (*n* = 12). Abbreviations: FDC (cefiderocol), IMI (imipenem), I/R (imipenem-relebactam), MEM (meropenem), M/V (meropenem-voborbactam), CTX (cefotaxime), CAZ (ceftazidime), CZA (ceftazidime-avibactam), C/T (ceftolozane-tazobactam), PIP (piperacillin), TZP (piperacillin-tazobactam), AZA (aztreonam-avibactam), CHL (chloramphenicol), CIP (ciprofloxacin), LVX (levofloxacin), CST (colistin), SXT (sulfamethoxazole-trimethoprim), AMK (amikacin). (**a**) consisting of *E. roggenkampii* (*n* = 44), *Enterobacter asburiae* (*n* = 3), and *Enterobacter soli* (*n* = 1). (**b**) consisting of *K. oxytoca* (*n* = 16) and *Klebsiella michiganensis* (*n* = 8).

All isolates were resistant to ureidopenicillins (e.g. piperacillin) and cephalosporins (e.g. cefotaxime, ceftazidime), whereas slightly lower resistance rates—ranging from 83.5–89.7%—were observed for β-lactam/β-lactamase inhibitor combinations, such as piperacillin-tazobactam, ceftolozane-tazobactam, and ceftazidime-avibactam (Fig. 1). Resistance to carbapenems was notably high, with rates of 77.3% for imipenem and 82.5% for meropenem. However, the combination of carbapenems with β-lactamase inhibitors significantly reduced resistance, with rates dropping to 32.9% for imipenem-relebactam and 10.1% for meropenem-vaborbactam. Importantly, all isolates were fully susceptible to aztreonam-avibactam.

Amikacin and trimethoprim-sulfamethoxazole exhibited comparatively lower resistance rates of 28.9% and 32.0%, respectively. In contrast, resistance to fluoroquinolones remained high, with rates of 86.6% for ciprofloxacin and 77.3% for levofloxacin. Resistance rates for chloramphenicol and colistin were also substantial, recorded at 75.3% and 59.8%, respectively.

Notably, three isolates from TCH1, TCH2, and TCH5—identified as *E. roggenkampii*, *S. marcescens*, and *E. asburiae*—were classified as PDR, exhibiting resistance to cefiderocol and the following tested antimicrobials: TZP, CTX, CAZ, CZA, C/T, IMI, MEM, AMK, CIP, LVX, SXT, ATM, CHL, and CST. However, these isolates remained susceptible to newer agents such as aztreonam-avibactam, although *E. roggenkampii* exhibited resistance to imipenem-relebactam and *E. asburiae* was resistant to meropenem-vaborbactam.

Furthermore, eleven *K. oxytoca* isolates from TCH5 and one *C. farmeri* isolate from TCH1 were classified as XDR, displaying susceptibility to amikacin and colistin. An *E. asburiae* isolate from TCH5 was also classified as XDR, showing susceptibility to aztreonam. Notably, all of these isolates were sensitive to the newer agents aztreonam-avibactam, imipenem-relebactam, and meropenem-vaborbactam.

### Clonal distribution and sequence types across hospital sites

From the 97 recovered isolates, 79 were selected for detailed characterization via WGS. Selection criteria included representing diverse phenotypic profiles, ensuring coverage of all sampling points to maximize temporal variability, and minimizing redundancy by excluding potential clonal isolates. The latter was particularly important to address possible biases introduced by pre-enrichment procedures during selective isolation from wastewater.

**Fig. 2:**
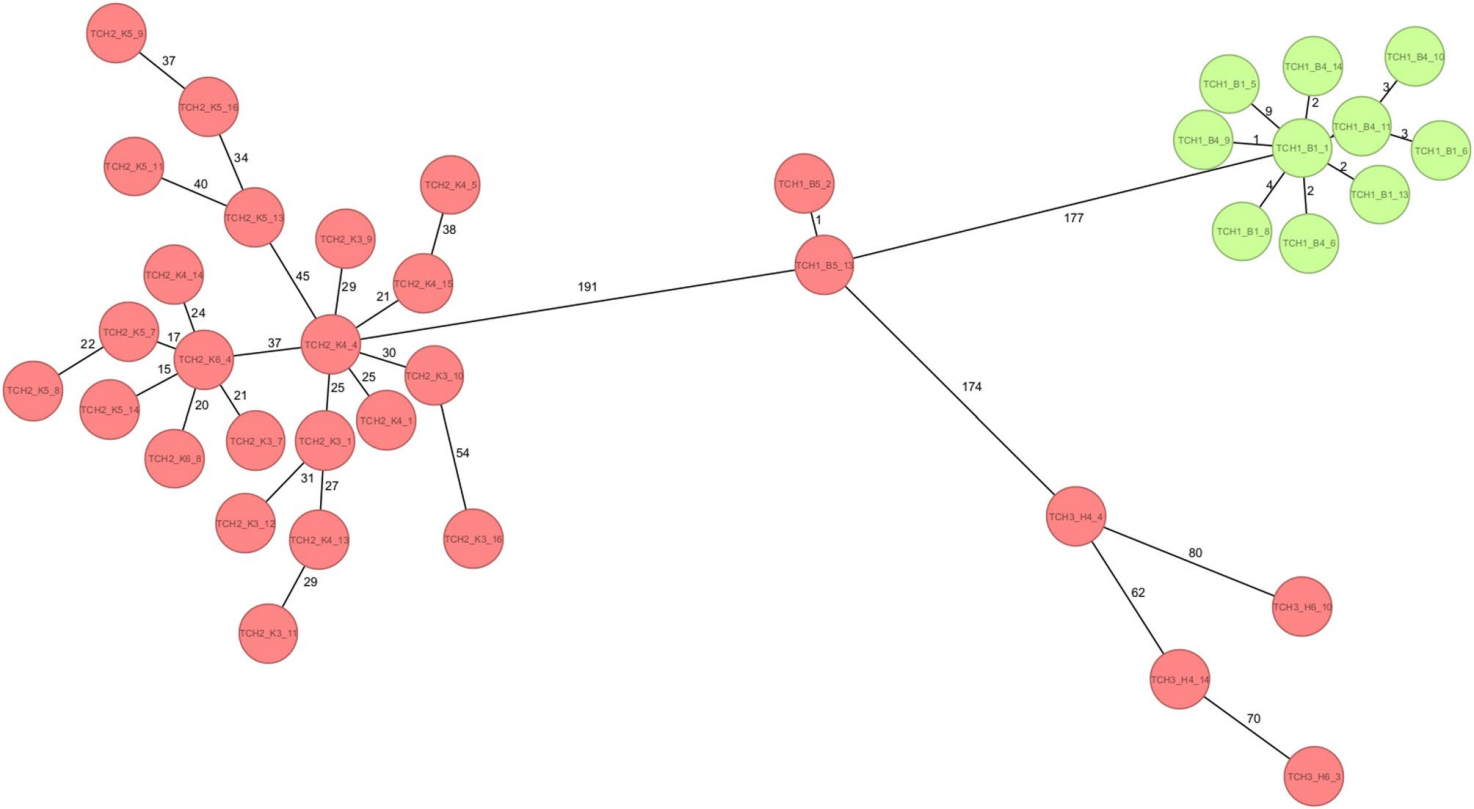
Minimum-spanning tree of *Enterobacter roggenkampii* (*n* = 38) based on cgMLST analysis (2,466 loci). Each circle represents an individual isolate; numbers on connecting lines indicate allelic differences. Red-filled circles denote isolates belonging to ST165, while green-filled circles represent isolates assigned to a novel sequence type.

At TCH1, all *C. farmeri* isolates (*n* = 7) were identified as belonging to sequence type (ST) 857. Clonal distributions of *E. roggenkampii* varied by site. At TCH1, a minority of isolates belonged to ST165 (16.7%, *n* = 2/12), while the majority (83.3%, *n* = 10/12) were non-typeable due to a nucleotide change in the *rplB* locus. cgMLST provided deeper insights into genetic relationships, confirming that the two ST165 isolates were clonal, differing by just one allele. In contrast, the *E. roggenkampii* isolates, representing a novel MLST type, differed from ST165 by 177 alleles and formed a distinct cluster with 1–9 allelic differences among themselves (Fig. 2). In comparison, TCH2 and TCH3 displayed greater clonal homogeneity, with all isolates at both sites belonging to ST165 (*n* = 22 in TCH2, *n* = 4 in TCH3). All *E. roggenkampii* isolates from TCH2 and TCH3 belonged to ST165 but showed substantial cgMLST allelic differences compared to the ST165 isolates from TCH1, highlighting the genetic diversity not captured by MLST.

**Fig. 3:**
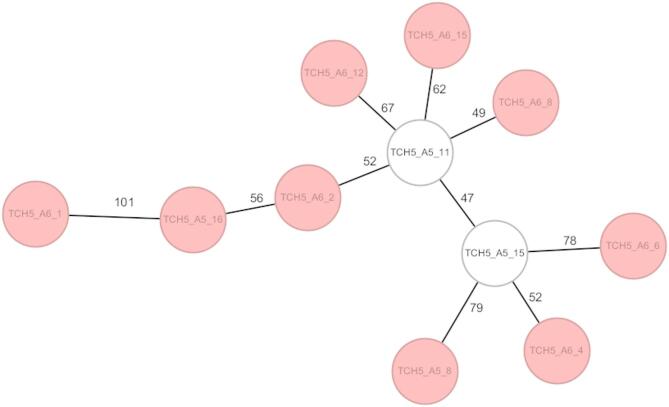
Minimum-spanning tree of *Klebsiella oxytoca* (*n* = 11) isolates from TCH5 based on cgMLST (2,538 loci). Each circle represents an individual isolate; numbers on connecting lines indicate allelic differences. Red-filled circles indicate isolates belonging to ST2, while unfilled (colorless) circles represent isolates assigned to a novel sequence type.

At TCH5, isolates from the *Klebsiella oxytoca* complex were predominantly identified as *K. oxytoca* ST2 (81.8%, *n* = 9/11). Two isolates were non-typeable using the classical MLST scheme based on seven housekeeping genes due to a single nucleotide change in the *phoE* locus. While the majority of isolates belonged to ST2, cgMLST revealed allelic differences ranging from 47 to 101, indicating notable genetic diversity within this sequence type and suggesting the presence of distinct sublineages (Fig. 3). In contrast, all isolates from TCH4 (*n* = 8) were identified as *K. michiganensis* ST35.

**Fig. 4:**
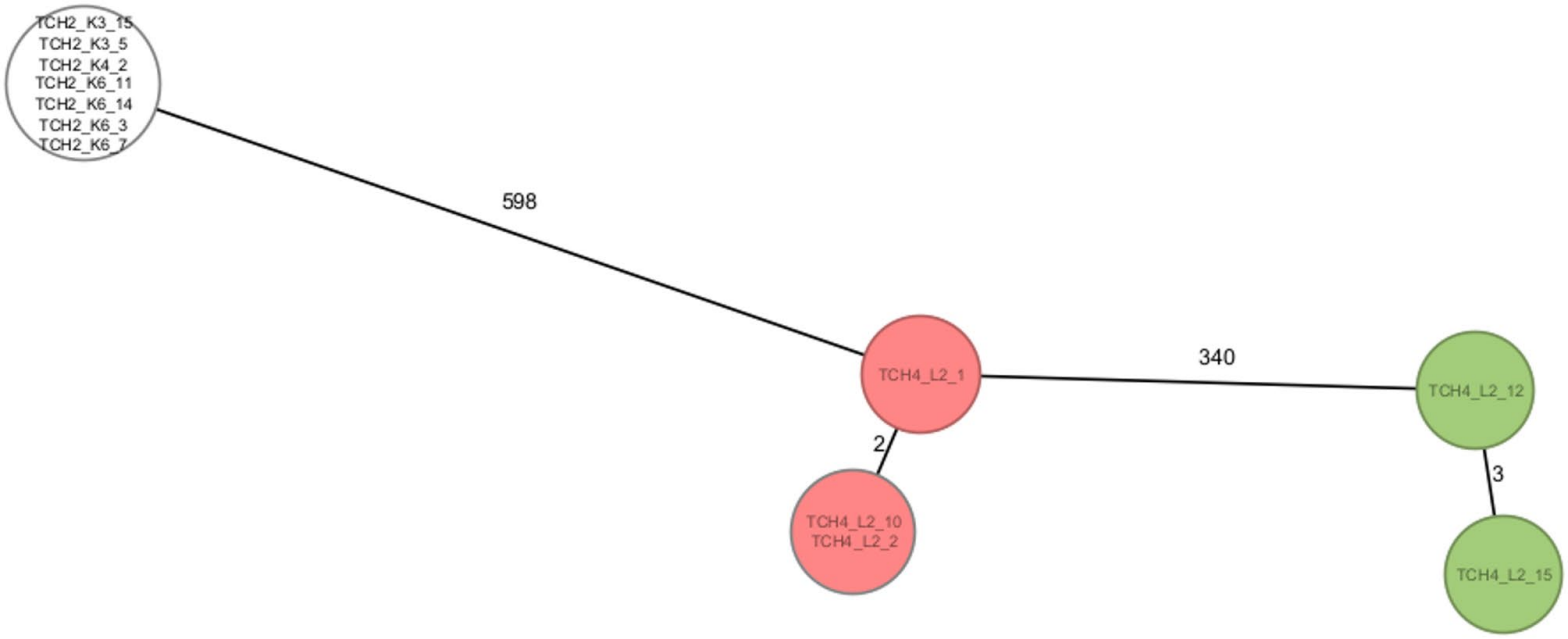
Minimum-spanning tree of *Serratia marcescens* (*n* = 12) based on cgMLST (2,682 loci). Each circle represents an individual isolate; numbers on connecting lines indicate allelic differences. Red-filled circles indicate isolates belonging to ST473, green-filled circles represent ST731, and unfilled (colorless) circles correspond to isolates assigned to novel sequence types.

*Serratia marcescens* isolates from TCH2 (*n* = 7) were entirely non-typeable by MLST, potentially indicating novel or uncharacterized lineages. cgMLST analysis further supported this notion, as the TCH2 isolates formed a distinct cluster with at least 598 allelic differences from the TCH4 isolates, suggesting a genetically separate lineage (Fig. 4). Conversely, *S. marcescens* isolates from TCH4 exhibited greater genetic diversity. MLST assigned 40% (*n* = 2/5) of the isolates to ST473 and another 40% to ST731, while one isolate remained non-typeable. However, cgMLST provided a more detailed resolution, revealing allelic differences ranging from 2 to 340 among the TCH4 isolates. While some formed closely related clusters (≤ 3 allelic differences), others showed substantial divergence, further emphasizing the ability of cgMLST to resolve genetic relationships at a much finer scale than classical MLST.

### Antimicrobial resistance genes and multi-drug potential

We identified distinct ARG profiles among the recovered cefiderocol-resistant bacterial species, with a notable prevalence of β-lactamase and carbapenemase genes. The detected ARGs and their combinations are summarized in Supplementary Table S1. In brief, and focusing solely on carbapenemase genes: *C. farmeri* (7/7) carried *bla*_NDM−1_ alone (*n* = 3) or in combination with *bla*_OXA−48_ (*n* = 4); *E. roggenkampii* (30/38) harbored either *bla*_NDM−1_ (*n* = 16) or *bla*_VIM−1_ (*n* = 14); all *K. oxytoca* isolates (11/11) co-carried *bla*_OXA−48_ and *bla*_VIM−1_; and *S. marcescens* (12/12) predominantly encoded *bla*_KPC−2_ (*n* = 9), with additional isolates carrying *bla*_VIM−1_ either alone (*n* = 1) or in combination with *bla*_OXA_ (*n* = 2). A detailed overview of the full resistome follows in the species-specific sections below.

*C. farmeri* (*n* = 7) displayed the most diverse genotypic resistance profile among the species, with ARG counts ranging from 6 to 22 per isolate. Prominent β-lactamase genes, including *bla*_SHV−12_, *bla*_TEM−1_, and *bla*_CTX−M−15_, were frequently identified, underscoring resistance across multiple β-lactams. Notably, one isolate from TCH1 harboured six distinct β-lactamase genes: *bla*_OXA−9_, *bla*_TEM−1_, *bla*_NDM−1_, *bla*_CTX−M−15_, *bla*_OXA−1_, and *bla*_SHV−12_. Beyond β -lactams, resistance was observed in quinolones (*qnrS1*), phenicols (*catB3*), macrolides (*mph(A)*) and aminoglycosides (e.g., *aac(6’)-Ib*, *aadA1* and *rmtC*).

*Enterobacter roggenkampii* (*n* = 38) isolates showed ARG counts from 4 to 19 per isolate, with β-lactam resistance largely attributed to *bla*_TEM−1_ and *bla*_CTX−M−15_, supporting broad resistance across β-lactams, including extended-spectrum cephalosporins. Significant ARGs outside of β-lactams included *aac(6’)-Ib* for aminoglycosides, *qnrB1* and *qnrB2* for quinolones, *catB3* for phenicols, *sul1* and *sul2* for sulfonamides, and *tet(A)* for tetracyclines. In the two *E. asburiae* isolates, 9 and 14 ARGs were identified, with carbapenemase genes *bla*_NDM−1_ and *bla*_VIM−1_ each present in separate isolates. Key β-lactamase genes in this species included *bla*_ACT−2_ and *bla*_OXA−1_, contributing to resistance against cephalosporins. Additional ARGs included *aac(6’)-Ib4* and *aadA1* for aminoglycosides, *qnrB1* and *qnrS1* for quinolones, *catA1* for phenicols, *sul1* for sulfonamides, and *tet(A)* for tetracyclines.

In the *K. oxytoca* complex (*n* = 19), ARG counts ranged from 7 to 17 per isolate. Relevant ARGs included *aac(6’)-IIc*, *aadA1*, *aph(3’’)-Ib*, *aph(3’)-Ia*, *aph(6)-Id* (aminoglycosides), *qnrS1* (quinolones), *catA1* (phenicols), *sul1* (sulfonamides), *dfrA1* (trimethoprim) and *tet(A)* (tetracyclines). Interestingly, all *K. michiganensis* isolates from TCH4 (*n* = 8) lacked carbapenemase-encoding genes. Six of these isolates displayed phenotypic susceptibility to ceftazidime-avibactam, imipenem, and meropenem, while all were resistant to cefiderocol. These isolates carried *bla*_SHV−12_ in combination with *emrD*, encoding a multidrug efflux pump.

*Serratia marcescens* (*n* = 12) exhibited one of the broadest ranges of ARGs, with counts ranging from 11 to 20 per isolate, highlighting its exceptionally high multidrug resistance potential. The presence of β-lactamase genes, including *bla*_TEM−1_, *bla*_SHV−12_, *bla*_OXA−1_, *bla*_OXA−2_, *bla*_ACC−1_, *bla*_SRT_, *bla*_CTX−M−9_ and *bla*_CTX−M−15_, contributed extensively to β-lactam resistance. Further resistance mechanisms involved *aac(6’)*,* aac(6’)-Ib*,* aac(6’)-Ib4*,* aadA1*,* aph(3’’)-Ib*,* aph(6)-Id* for aminoglycosides, *qnrB1* for quinolones, *catA1* for phenicols, *sul1* for sulfonamides, and *tet(41)* for tetracyclines, establishing *S. marcescens* as highly resistant across multiple antibiotic classes. Of particular importance is the presence of multiple efflux pump-encoding genes, including *sdeB*, *sdeY*, and *smfY*.

### Resistance to biocides and heavy metals

The analysis of resistance gene profiles revealed significant biocide and heavy metal resistance across bacterial isolates, underscoring their adaptability to environmental and anthropogenic stressors. The most prevalent biocide resistance gene, *qacEdelta1*, was detected in 63.3% (*n* = 50/79) of isolates, including *C. farmeri*, *E. roggenkampii*, *K. oxytoca*, and *S. marcescens*, indicating widespread resistance to quaternary ammonium compounds commonly used in disinfectants. *qacE* was found in 24.0% (*n* = 19/79) of isolates and frequently co-occurred with *qacEdelta1*, suggesting potential synergistic effects that enhance resistance. Notably, *S. marcescens* exhibited the highest diversity of biocide resistance genes, with up to six distinct genes per isolate, while *E. roggenkampii* consistently carried *qacEdelta1* and *qacE*, further highlighting species-specific adaptations to biocidal agents.

Heavy metal resistance was similarly widespread, with the genes *silA*, *arsC*, and *fief* identified in 97.5% (*n* = 77/79) of isolates, reflecting robust resistance to silver and arsenic. Complementary genes, such as *silR* and *silP*, were present in 83.5% (*n* = 66/79) of isolates. *K. oxytoca* complex isolates harboured the highest number of metal resistance genes, averaging 26.7 genes per isolate, followed by *E. roggenkampii* (20.1 genes) and *C. farmeri* (18.6 genes). *S. marcescens* displayed lower averages (7.8 genes) but still demonstrated notable variability. Species-specific patterns emerged, with *sil* and *ars* genes prevalent in *C. farmeri* and *Enterobacter* species, while *K. oxytoca* complex and *S. marcescens* exhibited similar profiles. Mercury resistance genes, such as *merE* and *merB*, were sporadically detected, adding a layer of defence in isolates with broader resistance capabilities. These findings underscore the resilience of bacterial communities in biocide- and metal-stressed environments, highlighting the potential challenges in healthcare and environmental settings where these selective pressures are prevalent.

### Virulence mechanisms: intrinsic and acquired factors

This study identified a wide range of virulence factors across bacterial isolates. Notably, the acquired factor *clpV* was detected in eight *K. michiganensis* isolates from TCH4. In contrast, none of the *Klebsiella* isolates exhibited the virulence-associated factors yersiniabactin (YbST), colibactin (CbST), aerobactin (AbST), or salmochelin (SmST), nor the regulatory genes *rmpA* and *rmpA2* (RmpADC) linked to the hypermucoid phenotype and enhanced capsule production.

However, intrinsic factors were prevalent across the investigated isolates, with *rcsB* (78 isolates) playing a key role in immune modulation and biofilm formation, and *ompA* (66 isolates) contributing to immune evasion and host cell adhesion. Key iron acquisition genes such as *entB* and *fepC* (66 isolates each) enhance survival in iron-limited environments, while *fliC* supports motility and tissue invasion. Additional factors contribute to adherence, antimicrobial activity, biofilm formation, effector delivery, invasion, and regulation, providing a comprehensive virulence toolkit. Notably, the absence of *tolC* renders the AcrAB-TolC efflux pump incomplete, potentially diminishing its role in resistance and virulence.

### Plasmid diversity and incompatibility types

Our analysis revealed notable variation in plasmid loads among species. *C. farmeri* exhibited the highest average plasmid load (56 plasmid incompatibility (Inc) types across seven isolates), followed by the *K. oxytoca* complex (137 Inc types across 19 isolates). *Serratia marcescens* and *E. roggenkampii* carried moderate loads, with 78 plasmid Inc types across 12 isolates and 199 plasmid Inc types across 38 isolates, respectively.

Across species, 38 unique plasmid Inc types were identified, with all detected Inc types listed in Supplementary Table S2. Of these, 18 were exclusive to a single species, indicating potential species-specific associations. Notable exclusive types included Col(IMGS31) in *C. farmeri*, IncFIB(pECLA) in *E. roggenkampii*, and IncFIB(pKPHS1) in *K. oxytoca*.

In contrast, 20 plasmid Inc types appeared across multiple species, suggesting their potential to facilitate gene transfer and adaptation across genera. ColRNAI, found in *C. farmeri*, *E. roggenkampii*, *K. oxytoca*, and *S. marcescens*, and Col440II, primarily associated with *E. roggenkampii* but also found in *C. farmeri* and *S. marcescens*, highlight adaptability and likely involvement in interspecies gene transfer.

Plasmids IncHI2A and IncHI2 were also present across multiple species, known for their association with antimicrobial and metal resistance, enhancing bacterial survival in challenging environments. Col440I was prevalent in *E. roggenkampii* and also appeared in *K. oxytoca* and *S. marcescens*, reflecting adaptability across genera.

Conversely, IncFIB(pECLA) and IncFII(pECLA) were exclusive to *E. roggenkampii*, indicating possible species-specific roles in resistance. IncFIB(K), primarily in *K. oxytoca* with occasional presence in *S. marcescens*, is strongly associated with *Klebsiella* but exhibits cross-genera adaptability.

Finally, IncX3 *showed* broad distribution across *E. asburiae*, *E. roggenkampii*, *K. oxytoca*, and *S. marcescens*, underscoring its versatility.

Detailed information on the phenotypic and genotypic characteristics of each isolate, along with its isolation source and date, is provided in Supplementary Table S3.

## Discussion

Cefiderocol-resistant bacteria in wastewater and aquatic environments remain largely underreported. Cimen et al.. (2023) reported two *E. cloacae* isolates from surface waters in Lower Saxony, Germany, one harbouring *bla*_OXA−181_ and the other *bla*_KPC−2_^12^. Similarly, Eger et al.. (2024) described two cefiderocol-resistant *E. coli* isolates from surface waters in Kumasi, Africa, both carrying *bla*_NDM−5_^13^. This limited number of reports may also reflect the fact that cefiderocol susceptibility testing is not routinely performed, as it requires the preparation of a specialized medium, adding complexity to the testing process^14^.

This study offers novel insights into the resistome and accessory genome features of cefiderocol-resistant Enterobacterales isolated from hospital wastewater at six German tertiary care centers. To the best of our knowledge, this is among the first reports to document the high ARG burden in cefiderocol-resistant XDR/PDR Enterobacterales from clinical wastewater in Germany.

The isolates exhibited species-specific resistance profiles, with a wide array of β-lactamase and carbapenemase genes. Notably, *Citrobacter farmeri* harbored up to 22 distinct ARGs, followed by *Serratia marcescens* (20 ARGs) and *Enterobacter roggenkampii* (19 ARGs), underscoring the substantial genetic potential for multidrug resistance in these environmental strains. Although clinical isolates with similarly high ARG burdens have been rarely documented, Cañada-García et al.. (2022) reported up to 20 ARGs in clinical carbapenemase-producing *K. pneumoniae* isolates from Spain^15^. Yao et al.. (2021) documented *C. freundii* and *Citrobacter portucalensis* isolates carrying 23 ARGs, including *bla*_VIM−2_ and *bla*_NDM−5_, from rectal swabs of colonized patients in Germany^16^. Jure et al.. (2021) described hypermucoviscous KPC-2-producing, carbapenem-resistant *K. pneumoniae* isolated from hospitalised patients in Argentina with 20 ARGs^17^. Carlsen et al.. (2022) reported *E. cloacae* complex ST24 isolates from hospital wastewater in Germany harbouring 20–21 ARGs, including combinations of *bla*_OXA−48_ with *bla*_VIM−1_, and *bla*_VIM−1_ with *bla*_VIM−2_. Additionally, *C. freundii* ST91 isolates were found to carry 20–22 ARGs, including the combination of *bla*_OXA−48_ with *bla*_VIM−1_^18^.

Sequence types identified here align with previously reported clinical and environmental sources, although reports on their occurrence remain limited for some. *K. oxytoca* ST2, commonly associated with human infections, has been reported across Europe, the USA, and Argentina^19^. *E. asburiae* ST162 has been identified in rectal swabs from patients at admission, while *E. roggenkampii* ST165 has been reported in a hematology unit in the Netherlands^20,21^. *K. michiganensis* ST35 has been recorded in the PubMLST database, with isolates originating from blood and stool samples in China, Austria, and the UK (accessed 2024-11-28). However, no records were found for *C. farmeri* ST857 or *S. marcescens* ST473 and ST731, highlighting a potential emergence of novel pathotypes.

The diversity of resistance determinants identified in the isolates of this study underscores the significant challenges posed by multidrug-resistant pathogens in both clinical and environmental settings. This highlights the substantial resistance burden carried by *Enterobacterales*, particularly *Citrobacter* spp. and the *Enterobacter cloacae* complex. Among these determinants, the diversity and co-occurrence of β-lactamases and carbapenemases are particularly noteworthy^22^. The identification of isolates harbouring up to six different β-lactamases exemplifies their remarkable adaptive capacity. Combinations of carbapenemase genes such as *bla*_NDM-1_, *bla*_VIM-1_ and *bla*_OXA-48_ together with ESBL determinants like *bla*_CTX-M-15_, *bla*_SHV-12_ and AmpC enzyme-encoding genes, are hypothesized to enhance resistance through synergistic hydrolytic activity, potentially further compromising cefiderocol efficacy. This extensive resistance is reflected in the high MICs observed in isolates carrying these combinations, emphasizing the urgent need for advanced diagnostic tools to detect and address such complex resistance mechanisms effectively. Lewis et al.. (2024) suggested that combining cefiderocol with novel β-lactamase inhibitors such as sulbactam, avibactam, and tazobactam emerges as a highly promising strategy, significantly enhancing cefiderocol’s activity against carbapenem-, cefiderocol-resistant *Enterobacterales*^23^. Our findings align with the study by Sader et al.. (2018)^24^, which reported high inhibition rates of aztreonam-avibactam against clinical *Enterobacterales* isolates from the United States carrying combinations of genes encoding metallo-β-lactamases and/or OXA-48-like enzymes. However, unlike Sader et al. (2018), who evaluated a broad set of carbapenem-resistant isolates, we specifically investigated aztreonam-avibactam susceptibility in a pre-selected population of cefiderocol-resistant isolates. This targeted approach provides a more focused insight into potential treatment options for infections caused by pathogens with resistance to multiple last-line agents, including cefiderocol, and highlights the continued therapeutic value of aztreonam-avibactam in such challenging cases.

While the co-occurrence of multiple carbapenemase types, such as metallo-β-lactamases and OXA-48-like enzymes, is generally rare in Europe^25^, exceptions have been observed. For instance, Bianco et al.. (2022) reported dual carbapenemases, such as NDM with OXA-48-like and VIM with OXA-48-like, in only 0.16–0.3% of 1,242 clinical carbapenemase-producing *Enterobacterales* (CPE) from Italy^26^. In Germany, *bla*_NDM-1_ in combination with *bla*_OXA-48_ was identified in 3.7% (*n* = 103/2,796) of CPE, while *bla*_OXA-48_ combined with *bla*_VIM-1_ was detected in 0.5% (*n* = 14/2,796) of CPE cases^27^. Higher rates were detected in wastewater isolates from German tertiary care hospitals, where 21.9% of *E. cloacae* complex (*n* = 7/32) and 22.2% of *S. marcescens* (*n* = 10/45) harboured combinations of *bla*_OXA-48_ with *bla*_VIM-2_, and *bla*_OXA-48_ with *bla*_GES-5_^18^.

In this study, *E. roggenkampii*, *C. farmer*,* S. marcescens* and *K. oxytoca* emerged as significant reservoirs of multidrug resistance, frequently carrying multiple carbapenemases. *C. farmeri* commonly harboured *bla*_VIM-1_ and *bla*_OXA-48_, while *K. oxytoca* predominantly carried *bla*_VIM-1_ and *bla*_OXA-48_. In contrast, *E. roggenkampii* displayed independent occurrences of *bla*_NDM-1_ and *bla*_VIM-1_, reflecting variability in gene co-occurrence patterns. Interestingly, *K. michiganensis* exhibited cefiderocol resistance despite lacking carbapenemases, likely mediated by *bla*_SHV-12_, *bla*_OXY-2-16_, and efflux pump activity (*emrD*,* oqxA*,* oqxB*), highlighting the importance of alternative resistance mechanisms^28^. Cefiderocol resistance in *E. cloacae* complex and *K. pneumoniae* can also arise from vertically inherited mechanisms, including mutations in siderophore receptors (*cirA*, *fiu*), porins (*ompK35*, *ompK36*, *ompK37*), and penicillin-binding protein 3 (PBP3), as well as increased efflux activity mediated by SugE and the heavy metal transporter ChrA, which impair antibiotic uptake or promote its extrusion^29–34^. However, these mechanisms are generally considered less relevant from an epidemiological standpoint due to their limited potential for horizontal dissemination. A direct comparison of nucleotide sequences between cefiderocol-resistant and -sensitive isolates, along with functional validation using knockout mutants or complementation assays, would be necessary to confirm the causal role of these mutations. Such analyses were beyond the scope of the present study but warrant further investigation.

Beyond β-lactam resistance, plasmid-encoded ARGs conferring resistance to aminoglycosides, quinolones, sulfonamides, and phenicols illustrate the genetic plasticity of these isolates and accumulation of resistance determinants via horizontal gene transfer^35^.

The diversity and abundance of plasmids among *Enterobacterales* highlight their role in ARG dissemination. Species-specific variations in plasmid loads, with *C. farmeri* and *K. oxytoca* harbouring the highest numbers of plasmid Inc types, highlight differential adaptability shaped by environmental pressures and horizontal gene transfer dynamics. Species-specific incompatibility types (e.g., Col(IMGS31)_1 in *C. farmeri* and IncFIB(pKPHS1) in *K. oxytoca*) suggest plasmid-host co-evolution, while widely distributed plasmids like ColRNAI and IncX3 facilitate interspecies ARG transfer, particularly in complex environments such as wastewater and clinical settings^36,37^. The association of high plasmid loads with extensive ARG diversity underscores their role in multidrug resistance. *Citrobacter farmeri* frequently carried plasmid-encoded carbapenemases (*bla*_NDM−1_, *bla*_OXA−48_) and β-lactamases (*bla*_TEM−1_, *bla*_SHV−12_, *bla*_CTX−M−15_), while *K. oxytoca* harboured similar resistance profiles. Notably, plasmid types IncHI2A and IncHI2, which harbour resistance genes for antibiotics, heavy metals and disinfectants/biocides, play a crucial role in bacterial survival under diverse environmental stressors^38^. The frequent co-localization of ARGs with biocide and heavy metal resistance genes drives co-selection and persistence in environmental reservoirs.

The adaptability of plasmids, such as IncX3, across species (e.g., *E. roggenkampii*, *K. oxytoca*, and *S. marcescens*), which impose minimal fitness cost on their bacterial hosts and exhibit high epidemic potential, significantly facilitates interspecies resistance transfer, posing a serious threat to treatment strategies^39^. These facts underscore the urgent need for effective interventions to curb the spread of plasmid-associated resistance.

Hospital wastewater further exacerbates these challenges, serving as a critical reservoir for extensively (pan)drug-resistant bacteria, as well as their associated ARGs^18,40,41^. Untreated discharge into municipal systems may facilitate the spread of resistant pathogens into natural ecosystems, possibly creating pathways for re-entry into humans and animals through water cycles. The co-occurrence of biocide and heavy metal resistance genes with ARGs on mobile elements amplifies co-selection under selective pressures^42^. The widespread use of biocides like quaternary ammonium compounds in healthcare and the presence of heavy metals such as silver and arsenic in wastewater further exacerbate resistance dynamics, reinforcing the need for interventions to mitigate these risks^43,44^.

In this study, several isolates were classified as PDR. However, susceptibility testing revealed that they remained susceptible to newer agents introduced since the PDR/extensively drug-resistant (XDR) definitions by Magiorakos et al.. (2012)^45^, such as imipenem-relebactam, meropenem-vaborbactam, and aztreonam-avibactam. This challenges the appropriateness of labelling these isolates as truly pandrug-resistant. The findings underscore a key limitation of the current PDR definition and highlight the need to reassess resistance classifications to reflect emerging treatment options. We propose describing such isolates as “XDR with susceptibility to newer agents,” offering a more accurate representation of their clinical relevance. Revising resistance definitions to incorporate new agents will ensure they remain aligned with evolving therapeutic practices and retain clinical utility.

While this study offers valuable insights, its focus on wastewater from six German tertiary care hospitals may limit the generalizability of the findings. Expanding the sample size and incorporating longitudinal sampling could provide a more comprehensive understanding of temporal trends and broader epidemiological patterns. Future research should aim to address these limitations through sustained monitoring, and broader geographic sampling.

Notably, some clinically common bacterial species frequently associated with cefiderocol therapy—such as *K. pneumoniae* and *E. coli*—were underrepresented in our wastewater-based sampling. This observation may point to species- or lineage-specific factors influencing resistance development, particularly under selective pressure from cefiderocol in the presence of carbapenemases. These dynamics warrant further investigation, particularly under controlled exposure conditions, to better understand the biological underpinnings of species-dependent resistance emergence.

In summary, this study has determined the widespread presence of cefiderocol-resistant bacteria in hospital wastewater, with resistant pathogens being detected in 50% of samples. The identification of PDR/XDR isolates underscores the critical challenge these pathogens pose by evading nearly all available treatment options. Hospital wastewater serves as a significant reservoir for high-risk bacterial clones and resistance determinants. Through untreated discharges, these reservoirs may contribute to the dissemination of resistant bacteria into broader ecosystems, potentially impacting public and environmental health. These findings underscore the need for continued investigation into the role of hospital effluents in antimicrobial resistance dissemination and may inform future strategies to mitigate environmental transmission pathways.

The extraordinary diversity of ARGs and plasmids identified in this study emphasizes the adaptability of these bacteria. The coexistence of multiple carbapenemases on multidrug-resistance plasmids amplifies resistance profiles, while the presence of biocide and heavy metal resistance genes highlights their resilience to both clinical and environmental pressures. Mobile genetic elements further enhance horizontal gene transfer, compounding the challenges of treatment and containment.

Preventive measures such as enhanced wastewater surveillance, stricter discharge regulations, or pre-treatment of clinical wastewater should be investigated. Coordinated public health strategies integrating improved wastewater management and cutting-edge therapeutic developments are imperative to address the growing threat of antimicrobial resistance effectively.

## Materials and methods

### Sample collection

The study was conducted in six tertiary care hospitals in Germany (TCH1-TCH6) across different federal states, previously described in^46^. These hospitals have capacities of 950–1500 beds and handle 220,000–490,000 patients annually. Their wastewater, ranging from 600 to 950 m³ per day, is discharged directly into municipal treatment plants without preliminary treatment. Qualified wastewater samples were collected in accordance with the DIN 38402-11:2009-02, with six biological replicates per hospital, totaling 36 samples. Samples were transported at 5 ± 2 °C and processed within 24 h in a centralized laboratory.

### Isolation, identification and susceptibility-testing of cefiderocol-resistant bacteria

The isolation of cefiderocol-resistant bacteria was carried out with modifications to the method described by^14^. Initially, 5 mL of wastewater was filtered through a 0.22 μm cellulose acetate membrane filter (GVS North America, Sanford, ME, USA). The filter was then transferred in 50 mL of iron-depleted cation-adjusted Mueller-Hinton broth, supplemented with 22.5 µg/mL CaCl_2_, 11.25 µg/mL MgSO_4_, and 70 µg/mL ZnSO_4_ according to Nordmann et al., 2012^47^ and Hackel et al.., 2019^48^. Additionally, 8 µg/mL of cefiderocol (Fetcroja; Shionogi & Co., Ltd, Japan), 5 µg/mL amphotericin B (Sigma-Aldrich, St. Louis, MO, USA), and 20 µg/mL of vancomycin (Sigma-Aldrich, St. Louis, MO, USA) were added to the broth to ensure selective growth conditions and to suppress the growth of non-target microbial species^14^.

The samples were incubated at 37 °C for 18–24 h with vigorous shaking at 170 rpm. Subsequently, suitable ten-fold dilutions were plated onto CHROMagar Orientation (Becton Dickinson GmbH, Germany) screening agar and incubated at 37 °C for 18–24 h. Presumptive colonies of target species, including *Escherichia coli*, *Proteus* spp., *Klebsiella* spp., *Enterobacter* spp., *Serratia* spp., *Citrobacter* spp., and *Pseudomonas aeruginosa*, were selected and sub-cultured on Columbia agar with 5% sheep blood (Becton Dickinson GmbH, Germany), then incubated at 37 °C for 18–24 h. The identification of the bacterial species was performed using MALDI-TOF MS (Bruker Daltonics GmbH & Co. KG, Germany).

Cefiderocol MIC values in the range of 0.03–32 µg/mL were determined using UMIC Cefiderocol (Bruker Daltonics GmbH & Co. KG, Germany). Subsequently, the antimicrobial susceptibility of cefiderocol-resistant isolates was assessed using microdilution with the Micronaut-S MDR MRGN-Screening system (Bruker Daltonics GmbH & Co. KG, Germany). The MICs for aztreonam (ATM), imipenem-relebactam (I/R), meropenem-vaborbactam (M/V), and aztreonam-avibactam (AZA) were determined using Liofilchem^®^ MIC-test strips (Liofilchem, Italy). The testing was conducted in accordance with the Clinical and Laboratory Standards Institute (CLSI) guidelines (M07-A10). Evaluation of the results was performed using the clinical cut-off values as per the EUCAST v15, effective from January 2025^49^.

Isolates were categorized as either extensively drug-resistant (XDR) or pandrug-resistant (PDR) based on the guidelines by Magiorakos et al.. (2012)^45^, but with adjustments in line with the most recent Expert Rules by the EUCAST v15. Following recommendations, cefoxitin (AmpC screening), fosfomycin (not recommended beyond *E. coli*), and tigecycline (no defined breakpoints) were excluded from susceptibility testing.

To categorize cefiderocol-resistant isolates as XDR or PDR, the following antimicrobial agents were evaluated: piperacillin-tazobactam (TZP), cefotaxime (CTX) and ceftazidime (CAZ), ceftazidime-avibactam (CZA) or ceftolozane-tazobactam (C/T), imipenem (IMI) or meropenem (MEM), amikacin (AMK), ciprofloxacin (CIP) or levofloxacin (LVX), sulfamethoxazole-trimethoprim (SXT), aztreonam (ATM), chloramphenicol (CHL), and colistin (CST).

### Genomic DNA extraction, whole-genome sequencing (WGS) and bioinformatic analysis

Genomic DNA was extracted from 1 mL overnight lysogeny broth culture using the PureLink Genomic DNA Mini Kit (Invitrogen, Darmstadt, Germany) per the manufacturer’s instructions. DNA quality and fragment length were evaluated using the TapeStation 2100 system (Agilent Technologies, Santa Clara, CA, USA), and DNA quantification was performed with the Qubit 1× dsDNA HS Assay Kit (Thermo Fisher Scientific, Waltham, MA, USA). Libraries were prepared using the Rapid Barcoding Kit (SQK-RBK114.24) and sequenced on a GridION platform equipped with an R10.4.1 Flow Cell (Oxford Nanopore Technologies, Oxford, UK) during a 48-hour sequencing run. Raw sequencing data were basecalled using the Dorado basecaller (v7.3.9), ensuring a minimum data yield of 400 Mb per strain.

The raw sequencing reads were assembled using the MiLongA pipeline (https://gitlab.com/bfr_bioinformatics/milonga). MiLongA integrates several bioinformatics tools for quality control and *de novo* assembly of long-read sequences, including flye (v2.9-b1768), porechop (v0.2.4), NanoFilt (v2.8.0), abricate (v1.0.1), platon (v1.6), NanoStat (v1.5.0), Kraken (v2.1.2), CheckM (v1.1.3) and minimap2 (v2.23-r1111). Following assembly, the genomes were analyzed using the BakCharak pipeline (https://gitlab.com/bfr_bioinformatics/bakcharak) to identify antimicrobial resistance genes (ARGs), virulence factors and plasmids. The database versions and configurations used in BakCharak included PlasmidFinder (v2021-03-27), VFDB (all set A, v2022-08-26), the NCBI AMRFinder database (v2024-01-31.1), PubMLST (v2023-01-15), and Platon (v1.5.0). Core-genome MLST (cgMLST) typing was performed for *Enterobacter roggenkampii*^50^, *Klebsiella oxytoca*, and *Serratia marcescens*^51^ using Ridom SeqSphere + v. 4.0^52^ software. The respective cgMLST schemes comprised 2,466, 2,538, and 2,682 target genes.

## Supplementary Information

Below is the link to the electronic supplementary material.

Supplementary Material 1

Supplementary Material 2

## Data Availability

The data for this study has been uploaded to the Sequence Read Archive (SRA) under accession number PRJNA1195545.
