# Supplementary material for: Cefiderocol-resistant pathogens in German hospital wastewater: a reservoir for multidrug resistance
Source: Sci Rep. 2025 Aug 27;15:31622. doi: 10.1038/s41598-025-17379-2 (PMC12391349; doi:10.1038/s41598-025-17379-2)
Supplement: Supplementary file 2 — Supplementary Material 2 [file 41598_2025_17379_MOESM2_ESM.docx]

**Supplementary Material**

**Cefiderocol-Resistant Pathogens in German Hospital Wastewater: A Reservoir for Multidrug Resistance**

Tim Erler, Alexander Hoch, Laura Carlsen, Jennifer Dengler, Jens Andre Hammerl, Andreas J. Stroehlein, Marc Hoffmann, Johannes K. Knobloch, Christoph Lübbert, Cihan Papan, Jonathan Schmid-Burgk, Thomas Schwanz, Janine Zweigner, Anurag Kumar Bari, Basil Britto Xavier, John W. A. Rossen, Nico T. Mutters, Mykhailo Savin

Corresponding author: Dr. Mykhailo Savin, Institute for Hygiene and Public Health, University Hospital Bonn, Venusberg-Campus 1, 53127 Bonn, Germany. Email: [michael.savin-hoffmeyer@ukbonn.de](mailto:michael.savin-hoffmeyer@ukbonn.de)

**Table S1** Antibiotic resistance genes and their combinations detected in target cefiderocol-resistant isolates (n = 79) recovered from hospital wastewaters.

|  | ***C. farmeri***  **(n = 7)** | ***E. cloacae* complex^1^**  **(n = 3)** | ***E. roggenkampii***  **(n = 38)** | ***K. oxytoca* complex^2^**  **(n = 19)** | ***S. marcescens***  **(n = 12)** |
| --- | --- | --- | --- | --- | --- |
| **Aminoglycoside-quinolones** | aac(6')-Ib-cr5 (100.0%) | aac(6')-Ib-cr5 (100.0%) | aac(6')-Ib-cr5 (100.0%) | n.d. | aac(6')-Ib-cr5 (100.0%) |
| **Aminoglycosides** | aac(6')-Ib, aadA1, rmtC (62.5%); aac(3)-IIe, aac(6')-Ib, aadA1, aadA2, aph(3'')-Ib, aph(6)-Id (12.5%); aac(3)-IId, aac(6')-II, aadA1, aph(3'')-Ib, aph(3')-Ia, aph(6)-Id (12.5%); rmtC (12.5%) | aadA1 (50.0%); aac(6')-Ib4, aadA1 (50.0%) | aac(6')-Ib4, aadA1 (56.0%); aac(3)-IIe, aadA1, aph(3'')-Ib, aph(6)-Id (36.0%); aac(6')-Ib3 (8.0%) | aac(6')-IIc, aadA1, aph(3'')-Ib,aph(3')-Ia, aph(6)-Id (52.6%); aph(3')-Ia (42.1%); aac(6')-IIc, aadA1, aph(3')-Ia (5.3%) | aac(3)-IId, aac(6'), aadA2, ant(2'')-Ia, aph(3'')-Ib, aph(6)-Id (33.3%); aac(3)-IId, aac(6'), aph(3'')-Ib, aph(6)-Id (25.0%); aac(6'), aac(6')-Ib4, aph(3'')-Ib, aph(6)-Id (16.7%); aac(6'), aac(6')-Ib, aac(6')-Ib4, aadA1, aph(3'')-Ib, aph(6)-Id (16.7%); aac(6'), aac(6')-Ib4, aadA1, aph(3'')-Ib, aph(6)-Id (8.3%) |
| **β-Lactams** | blaOXA-9, blaTEM-1 (85.7%); blaOXA (14.3%) | n.d. | blaTEM-1 (92.3%); blaOXA, blaTEM-1 (7.7%) | n.d. | blaTEM-1 (44.4%); blaTEM-1, blaTEM-2 (33.3%); blaOXA-2 (22.2%) |
| **Bleomycin** | ble (100.0%) | ble (100.0%) | ble (100.0%) | n.d. | n.d. |
| **Carbapenems** | blaNDM-1, blaOXA-48 (50.0%); blaNDM-1 (50.0%) | blaNDM-1 (50.0%); blaVIM-1 (50.0%) | blaNDM-1 (53.3%); blaVIM-1 (46.7%) | blaOXA-48, blaVIM-1 (100.0%) | blaKPC-2 (75.0%); blaOXA, blaVIM-1 (16.7%); blaVIM-1 (8.3%) |
| **Cephalosporins** | blaSHV-12 (71.4%); blaCTX-M-15, blaOXA-1,blaSHV-12 (14.3%) | blaACT-2, blaOXA-1 (100.0%) | blaMIR-11 (60.5%); blaCTX-M-15, blaMIR-11, blaOXA-1 (21.1%); blaMIR-11, blaOXA-1 (10.5%); blaCMY-6, blaMIR-11 (5.3%); blaCTX-M-15, blaMIR-11 (2.6%) | blaOXY-2-16 (57.9%); blaOXY, blaSHV-12 (42.1%) | blaSHV-12, blaSRT (25.0%); blaOXA-1, blaSRT (16.7%); blaSRT (16.7%); blaCTX-M-9, blaSHV-12, blaSRT (16.7%); blaACC-1, blaSRT (16.7%); blaCTX-M-9, blaSRT (8.3%) |
| **Colistin** | n.d. | n.d. | n.d. | n.d. | mcr-9.2 (50.0%); mcr-9.1 (50.0%) |
| **Efflux** | n.d. | n.d. | n.d. | emrD (100.0%) | sdeB, sdeY, smfY (91.7%); sdeB,sdeY (8.3%) |
| **Fosfomycin** | n.d. | fosA (100.0%) | fosA (100.0%) | n.d. | n.d. |
| **Macrolides** | mph(A) (100.0%) | n.d. | n.d. | n.d. | mph(A) (100.0%) |
| **Phenicol-quinolones** | oqxB (100.0%) | oqxA, oqxB (100.0%) | oqxA, oqxB (100.0%) | oqxA, oqxB (94.7%); oqxB (5.3%) | n.d. |
| **Phenicols** | catA2, catB3 (50.0%); catB3, floR (50.0%) | catA1, catB3 (100.0%) | catA1, catB3 (69.2%); catB3 (30.8%) | catA1 (100.0%) | catB3 (50.0%); catA1 (50.0%) |
| **Quinolones** | qnrS1 (100.0%) | qnrB1 (50.0%); qnrS1 (50.0%) | qnrB1 (52.0%); qnrB2 (48.0%) | qnrS1 (100.0%) | qnrA1 (60.0%); qnrB2 (40.0%) |
| **Rifamycin** | arr-3 (100.0%) | n.d. | n.d. | n.d. | arr-3 (100.0%) |
| **Streptothricin** | n.d. | n.d. | n.d. | sat2 (100.0%) | n.d. |
| **Sulfonamides** | sul1 (75.0%); sul1, sul2 (25.0%) | sul1 (100.0%) | sul1 (64.0%); sul2 (36.0%) | sul1 (100.0%) | sul1 (100.0%) |
| **Tetracyclines** | tet(31) (100.0%) | tet(A) (100.0%) | tet(A) (100.0%) | tet(A) (100.0%) | tet(41) (100.0%) |
| **Trimethoprim** | dfrA12 (50.0%); dfrA1, dfrA15 (50.0%) | dfrA14 (100.0%) | n.d. | dfrA1 (100.0%) | dfrA19 (50.0%); dfrA16 (50.0%) |

^1^ *E. soli* (n = 1) and *E. asburiae* (n = 2)

^2^ *K. oxytoca* (n = 11) and *K. michiganensis* (n = 8)

Abbreviation: n.d., not detected

**Table S2** Plasmid incompatibility summary per species.

| **Species** | **Plasmid incompatibility Summary** |
| --- | --- |
| *C. farmeri*  (n = 7) | IncFIB(pB171) (7); IncFIA(HI1) (7);  IncFII(Yp) (7);  RepA(pKPC-CAV1321) (7);  pENTAS02 (7); ColRNAI (7);  Col440II (6);  IncL/M(pOXA-48) (4); Col(IMGS31) (3); IncX5(2) (3);  Col(IRGK) (3);  IncFIB(Mar) (1);  IncQ2 (1); IncHI1B(MAR) (1) |
| *E. roggenkampii*  (n = 38) | Col440II (27); IncFIB(pECLA) (24); IncFII(pECLA) (24); ColRNAI (22); Col440I (15); IncN (14); IncFII(Yp) (12); IncX3 (10); RepA(pKPC-CAV1321) (9); IncHI2 (9); IncHI2A (9); Col(MGD2) (8); repA(pKPC-2) (4); IncFIB(pQil) (4); IncHI1B(MAR) (3); IncFIB(Mar) (3); IncA/C2 (2) |
| *K. oxytoca* complex  (n = 19) | IncFIB(K) (19); I  ncFII(p14) (19); ColRNAI (14); IncFIB(pKPHS1) (11);  IncHI2A (11); IncHI2 (11);  IncL/M(pOXA-48) (11); RepA(pKPC-CAV1321) (11); IncHI1A(CIT) (8); IncHI1B(CIT) (8); IncX3 (8); Col440I (6) |
| *S. marcescens*  (n = 12) | IncN (7); pSM22 (7);  RepA(pKPC-CAV1321) (6); IncHI2 (6); IncHI2A (6); Col(MGD2) (6);  Col440I (5); Col(Ye4449) (4);  Col440II (4); ColRNAI (4); IncX5 (2) (3); IncFIB(K) (3); IncFII(pRSB107) (3);  pENTAS02 (3); IncQ2 (3); IncX3 (2); FII(pBK30683) (2);  IncFII(Yp) (2); IncL/M(pMU407) (1); IncFII(pCRY) (1) |
